# Risk aversion, trust in institutions and contingent valuation of healthcare services: trying to explain the WTA-WTP gap in the Dutch population

**DOI:** 10.1186/s12962-021-00281-9

**Published:** 2021-05-05

**Authors:** Jesús Martín-Fernández, Ángel López-Nicolás, Juan Oliva-Moreno, Héctor Medina-Palomino, Elena Polentinos-Castro, Gloria Ariza-Cardiel

**Affiliations:** 1 Family and Community Medicine Teaching Unit Oeste, Primary Care Management, Madrid Health Service, Móstoles, Madrid Spain; 2 Faculty of Health Sciences, Universidad Rey Juan Carlos, Alcorcón, Madrid Spain; 3 Research Network in Health Services and Chronic Diseases (REDISSEC), Madrid, Spain; 4 Economics Department, Faculty of Business Science, Universidad Politécnica de Cartagena (UPCT), Cartagena, Murcia Spain; 5 Department of Economic Analysis and Finance, Faculty of Law and Social Sciences in Toledo, University of Castilla La-Mancha, Toledo, Spain; 6 Stichting Medina Care, Rotterdam, The Netherlands; 7 Primary Care Research Unit, Primary Care Management, Madrid Health Service, Madrid, Spain

**Keywords:** Health services research, Contingent valuation, Economics, Patient preferences, Risk-taking, Trust

## Abstract

**Background:**

The preferences of citizens are a basic element to incorporate into the decision-making process when planning health policies. Contingent valuation (CV) is a common method for calculating the value for citizens that new technologies, interventions, and the provision of services or policies have. However, choosing the correct CV tool may not be a neutral decision. This work aims to assess the substitution of a healthcare service by comparing valuation differences between the willingness to pay (WTP) for the maintenance of the service versus the willingness to accept compensation (WTA) for its substitution, both of which are related to subject characteristics, with a particular focus on trust in institutions and risk aversion.

**Methods:**

A CV study was designed to study Dutch population preferences when physician assistants replace anaesthesiologists. Differences between the distributions of WTA and WTP were compared through full decomposition methods, and conditional quantile regression was performed.

**Results:**

Nearly two-thirds of surveyed citizens expressed null values for WTA and WTP. The other third systematically reported a value of WTA higher than that of WTP, which increased further with lower income and the possible presence of a strategic bias. In contrast, being more than 65 years old, having trust in government, and preferring anaesthesiologists decreased the WTA-WTP difference. Risk aversion had no clear association with the WTA-WTP gap.

**Conclusions:**

Known differences between the perceived value of health services from the perspective of gains and losses could be related to people’s characteristics. Trust in government but not aversion to risk was related to the WTA-WTP differences. Identifying a profile of citizens who are averse to losing health services should be considered when designing and implementing health services or interventions or making disinvestment decisions.

**Supplementary Information:**

The online version contains supplementary material available at 10.1186/s12962-021-00281-9.

## Background

Good governance for health positively influences all the functions of the health system, improving its performance and, ultimately, improving the health outcomes and well-being of the population [1]. Understanding the value placed on different healthcare services and discovering people’s preferences so as to shape health policy is an essential task for good governance, which has been shown to improve healthcare efficiency and quality [2]. The economics discipline proposes several methods for attributing a value to goods or services even when they are not exchanged in a real market. One of them is contingent valuation (CV), a method well-grounded in economics theory that assumes that personal preferences can be interpreted in the form of a utility function, where two states (initial and final) can be compared through the changes in the level of utility [3].

Services provided by healthcare systems are changing even as these systems face funding shortfalls across the world [4], and little is known about citizens’ preferences about these changes. Regarding public healthcare systems, governments employ multiple strategies to face funding needs [5], one of which is disinvestment: “the processes of withdrawing health resources from existing healthcare practices that are deemed to deliver little or no health gain for their cost” [6]. The literature shows a number of different forms of healthcare disinvestment, including full withdrawal or decommissioning, retraction, restriction, and substitution [4]. Substitution refers to processes in which an intervention, treatment, or practice is replaced by another one that is considered to be more efficient. A specific kind of substitution is that related to different skills, for example, substituting the performance of a task from one particular professional to another who is less specialized but can be more efficient [7].

European health systems have suffered a series of changes in the context of the economic crises at the beginning of this century. The Dutch health system has undergone deep reforms in the last 15 years. The most remarkable reform was implemented in 2006. A single health insurance scheme with managed competition was introduced [8]. The aims of these reforms were to promote efficiency, reduce central governance and improve access at acceptable social costs. Substitution and transfer of tasks from medical to nursing professionals was proposed as a relevant trend. We identified this trend as a typical sort of disinvestment strategy. The physician assistant (PA) profession development in the Netherlands has experienced remarkable legal changes. The 2012 law for PAs provisionally enabled them to practice independently of medical supervision. After a 5-year pilot program, the Dutch parliament, amended the Healthcare Professionals Act (Wet BIG) on July 1, 2018, granting PAs full independence in diagnosing, initiating treatments and performing medical procedures [9], and they are now allowed to deliver anaesthesia procedures such as catheterizations, drug prescription, pre-anaesthesia screening, and post-surgery follow-up, without the supervision of a medical doctor trained in anaesthesia (MDA). However, little evidence is available about the cost-effectiveness of such substitutions [10–12], and many questions about how patients value them are unresolved yet.

Citizens’ assessment of this sort of disinvestment strategy could be approached through the CV methodology. Citizens could be asked about the maximum monetary amount a person would be willing to pay for maintaining the service, namely, his/her willingness to pay (WTP). Alternatively, a person can be asked about their minimum willingness to accept compensation (WTA), which is the minimum compensation the same person would require for the change of the service (the substitution), so that the perceived utility for the subject remains constant with the change.

Under the Hicksian welfare theory, the WTA and WTP for a good or service should be similar for the same subject [13, 14]. However, it has been shown that observed WTA values are consistently higher than the WTP in the field of healthcare services [15–20], irrespective of the method used to evaluate them [21]. This gap has been consistently observed in other fields but is greater for non-market goods [22–24].

Several explanations for this disparity have been suggested within the neoclassical economics framework, such as “income effect” and transaction costs [25], the absence of substitutes [26, 27], and hypothetical biases where the less information there is about the valued good or the higher the costs of information are, the greater the WTA-WTP difference [28, 29]. Other alternative explanations about these differences challenge the normative framework of neoclassical economics. Prospect theory proposes the existence of an “endowment point”, which acts like the point of reference for the loss and gain, and valuations of gains and losses are always relative to it; losses are valued more heavily than gains, and the valuation function exhibits diminishing marginal valuation the further away from the reference point one gets [30]. Prospect theory appears to offer an excellent description of behaviour in experimental settings, predicting WTA-WTP differences [31]. It has been shown how this difference is increased in the presence of risk aversion [30, 32, 33], and it has been proposed that the former difference could also increase when trust (between buyers and sellers) decreases [33]. Citizens’ trust in institutions has been demonstrated to be a critical factor in determining their WTP [34–36] and is also related to their expressed WTA [37]. Moreover, higher levels of trust have been correlated with smaller risk perceptions [38]. Therefore, risk aversion and trust in institutions could be important characteristics for explaining the WTA-WTP gap.

It is important to keep studying the WTA-WTP difference, in this case for healthcare goods and services. Assessing healthcare strategies from different perspectives (gains and losses, WTP and WTA) is justified for understanding the preferences of citizens, and it has important implications for healthcare decision-making. If WTA is larger than WTP, different cost effectiveness thresholds should be used for decisions on stopping services or healthcare interventions, compared with decisions on starting new interventions, so the cost-effectiveness ratio should probably be significantly less favourable for disinvestment strategies than for starting a new intervention [24, 39, 40].

This paper aimed to apply CV techniques to assess the disparities between WTA and WTP expressions for the substitution of MDAs by PAs made by the Dutch government and to analyse the influence of personal characteristics and sociocultural values, especially trust in institutions and risk aversion, on these disparities.

## Methods

### Design

CV analysis (ex-ante perspective) was used to compare WTP and WTA for avoiding through payment or being compensated, respectively, for the substitution of a medical doctor trained in anaesthesia (MDA) by a physician assistants (PA) in anaesthetic procedures, as proposed by the Dutch government.

### Sample collection

The data were obtained from the University of Tilburg, The Netherlands, from CentERpanel, a web-based survey system based on demographic data from the Netherlands Central Bureau of Statistics. The panel consists of more than 2000 households representative of the Dutch population in the majority of dimensions, such as gender, age, province of residence, or income. Each panellist received a small compensation for every completed questionnaire which was paid out, either in cash or in the form of a donation to a charity or a state lottery, as the household preferred [41].

### Clinical scenario

Health insurance is compulsory in the Netherlands. It is financed mainly (72%) from citizens' contributions, with an additional 13% from general taxes. Adults pay a community premium to their insurer (the government contributes the premium for children), plus an income-related premium to a central fund which is redistributed among insurers according to risk. The first €385 (2019) of healthcare expenses in a given year must be paid out of pocket (except for general practitioners’ visits, maternity care and home nursing care). After having spent that amount (plus any voluntary deductibles), insurance takes over [8]. The clinical scenario was presented in this framework. After having read the descriptions for an MDA, a PA, and anaesthesia, participants were presented a scenario in which they were supposed to be a 55-year-old person in need of a rectosigmoid resection due to cancer of the large intestine. The type of procedure and age were chosen because these are associated with a 30-day surgery mortality risk, technically known as the Risk Quantification Index (RQI) [41], as was explained to the participants. For the sake of clarity, the RQI was expressed both in terms of percentage and ratio and was set at 0.5% or 1 in 200 patients for the described procedure, respectively.

The clinical scenarios are described in Additional file 1.

### Main variables and data collection

The WTP for maintaining the service and WTA for being compensated for the substitution were estimated through an open-ended iterative bidding system. Values of WTA and WTP were randomly generated by Blaise® software (University of Tilburg, The Netherlands) using an unfolding brackets algorithm that started at the first randomly generated value card for WTA and WTP. If the participant did not accept to pay the offered monetary value, they were then asked whether they would agree to pay the next lower value, but if they had expressed a positive willingness to pay, they were asked about their WTP for the next higher value. For eliciting the WTA the process was the reverse. If the highest value on the card was reached, the interviewee was allowed to offer an open higher value. This process was repeated until the participant indicated they were sure of their choice of value card. For WTA and WTP, the offered (€) value cards were 0, 25, 50, 100, 200, 400, 600, 800, 1000, 1500, 2000, and 3000. The obtained values were limited to €50,000, as this point represented the maximum selected by 99.5% of the interviewees.

The independent variables were sorted into the following categories: risk attitude, sociodemographic characteristics, health needs, use of services, and sociocultural values and beliefs (preferences and trust in the government or in the service providers).

Risk attitude was evaluated through the subjective perception of the interviewees. The subjects were asked their preference on a scale where 1 represented the highest risk aversion and 10 the highest risk inclination.

The sociodemographic characteristics collected from the participants were age (under or over 65 years), marital status (married or living with a couple), gender, level of education (low, medium, or high according to the ISCED classification) [42], and social class (high, medium-high, medium, medium-low, low). These characteristics were known for each person since they were included in the panel.

Health needs and use of services were measured by the number of visits to the family physician or other specialists during the last month, history of surgical interventions, and self-perceived health status on a Likert scale (from 1 being the worst to 5 being the best condition).

Preferences for an anaesthesiologist vs. a PA were elicited by a direct question (MDA, PA or indifferent). Trust in government or insurance companies was measured using two identical Likert scales where 1 stood for the lowest level of trust and 10 for the highest. To refine the CV answers, the subjects were asked about the possibility of having introduced a strategic bias in their response via a direct question: “You might be tempted to answer that you would be willing to pay very little or nothing for a service or good that you know is very important to you; by naming a very low price you would expect that the government might end up making that good or service free for all. How much do you think this applies in your case?”, with 1 = I completely disagree and 10 = I completely agree.

### Modelling and hypothesis

The following null hypotheses were to be tested:

H0i: There are no differences between the WTP and WTA distributions

This hypothesis must be rejected before testing the remaining ones.

H0_1: there are no significant differences between WTA and WTP by age and sex.

H0_2: there are no significant differences between WTA and WTP by educational level, social class and income level.

H0_3: greater risk aversion is not associated with greater differences between WTA and WTP.

H0_4: greater trust in institutions is not associated with smaller differences between WTA and WTP.

To test these hypothesis, following a descriptive analysis, full decomposition methods were employed to analyse differences between the distributions of WTA and WTP as proposed by Chernozhukov et al*.* [43]. Specifically, the conditional distributions of the WTP and WTA were modelled, and estimates were obtained using quantile regression for testing hypothesis H0_1 to H0_4 [44, 45].

Given the distributions F_WTP_(X,β_WTP_) and F_WTA_(X,β_WTA_) for WTP and WTA, respectively, expressed in terms of their dependence on subject characteristics (X) and a set of parameters (β), the difference between WTP and WTA can be represented as a function G that varies with the difference between the two sets of parameters and the subject characteristics:

1 $${F}_{WTP}\left({X,\beta }_{WTA}\right)- {F}_{WTA}\left({X,\beta }_{WTA}\right)=G( {\beta }_{WTP}-{\beta }_{WTA},X)$$

The methods proposed by Chernozhukov et al*.* [43] were applied to carry out a series of statistical tests for the WTP-WTA differences. Additionally, we considered it of interest to study the impact of some covariates on the differences between WTP and WTA at different points across their distributions. Thus, for the τ-th quantile of a given distribution, Q τ (.), the following specifications for the conditional quantile functions were employed:

2 $${Q}^{\tau }\left(WTP\right)={\beta }_{1}^{\tau }+\sum_{k=1}^{k}{\beta }_{k}^{\tau }{x}_{k}+{u}^{\tau }$$

3 $${Q}^{\tau }\left(WTA\right)={\gamma}_{1}^{\tau }+\sum_{k=1}^{k}{\gamma}_{k}^{\tau }{x}_{k}+{\varepsilon }^{\tau }$$

where ${\beta }_{1}^{\tau }$ and ${\gamma}_{1}^{\tau }$ are the intercept terms, ${\beta }_{k}^{\tau }$ and ${\gamma}_{k}^{\tau }$ are the parameters associated with subject characteristics, ${u}^{\tau }$ and ${\varepsilon }^{\tau }$ are stochastic errors, and τ equals 0 ≤τ ≤1.

Given a consistent estimation of the parameters for these models (2, 3), the following can be written:

4 $${Q}^{\tau }\left(WTP\right)={{\widehat{Q}}^{\tau } \left(WTP\right)+{\widehat{u}}^{\tau }=\widehat{\beta }}_{1}^{\tau }+\sum_{k=1}^{k}{\beta }_{k}^{\tau }{\overline{x}}_{k}+{\widehat{u}}^{\tau }$$

5 $${Q}^{\tau }\left(WTA\right)={{\widehat{Q}}^{\tau } \left(WTA\right)+{\widehat{\varepsilon }}^{\tau }=\widehat{\gamma}}_{1}^{\tau }+\sum_{k=1}^{k}{\gamma}_{k}^{\tau }{\overline{x}}_{k}+{\widehat{\varepsilon }}^{\tau }$$

where ($\overline{x}$) stands for the mean values of the explanatory variables. The difference is then equal to the following:

6 $${Q}^{\tau }\left(WTP\right)- {Q}^{\tau }\left(WTA\right)= \left[{\widehat{Q}}^{\tau } \left(WTP\right)-{\widehat{Q}}^{\tau } \left(WTA\right)\right]+\left[{\widehat{u}}^{\tau }-{\widehat{\varepsilon }}^{\tau }\right]$$

where the first element to the right of the equation is the explained part and the second element is a random error. The explained part can be decomposed as:

7 $${\widehat{Q}}^{\tau } \left(WTP\right)-{\widehat{Q}}^{\tau } \left(WTA\right)={ \widehat{\beta }}_{1}^{\tau }-{\widehat{\gamma}}_{1}^{\tau }+\sum_{k=1}^{k}( {\beta }_{k}^{\tau }-{\gamma}_{k}^{\tau }{) \overline{x}}_{k}$$

where the left part of the equation is the difference between the intercept terms, and the right part is the contribution of the explanatory variables to the model. The contribution to the absolute difference between WTP and WTA for each explanatory variable can be calculated through the relationship:

8 $$\frac{( {\beta }_{k}^{\tau }-{\gamma}_{k}^{\tau }{) \overline{x}}_{k}}{{\widehat{Q}}^{\tau } \left(WTP\right)-{\widehat{Q}}^{\tau } \left(WTA\right)}$$

where standard errors were calculated using bootstrapping techniques.

### Procedures

To specify the models, included explanatory variables were clustered in the following categories: sociodemographic (gender, being older than 65 years, being married, social class, and income), health needs and use of services (self-stated health status, number of visits to the healthcare system), sociocultural values and beliefs (preferences about the provided service, former surgeries, trust in the government, trust in the insurance company, and strategic bias), and self-stated aversion to risk. Use of health services, self-stated health status, and trust in insurance companies were omitted from the explanatory models since they did not improve the fit. Aversion to risk was dichotomized (subjects with scores below the scale median were classified as risk averse) to obtain the best model performance after testing other possibilities. A certain degree of collinearity was observed between social class and educational level, which made us choose the former as an explanatory variable.

Stata® 14 software was used for calculations.

## Results

An online survey was launched in three periods, from May 23 to 27, from May 30 to June 3 and from June 27 to July 1, 2014, to 2822 respondents available on CentERpanel at the time of the study. A total of 1905 (67.5%) surveys were finally completed. No significant differences were found between respondents and nonrespondents in terms of age, gender, educational level or place of residence.

Tables 1 and 2 describe the characteristics of the included subjects.

**Table 1:** Characteristics of participants

	N	Measurement	Values (95% CI)
Socio-demographic			
Age	1905	> 65 years	26.4% (24.4–28.4%)
Gender	1905	Women	49.4% (47.2–51.7%)
Married	1905	Yes	76.9% (75.0–78.8%)
Educational level	1905	Low (ISCED 1)	25.9% (23.9–27.8)
		Medium (ISCED 2,3,4)	29.4% (27.3–31.4%)
		High (ISCED 5,6)	44.7% (42.5–47.0%)
Social class	1897	High	5.0% (23.0–27.0%)
		Medium-high	33.2% (31.1–35.3%)
		High	24.9% (22.9–26.8%)
		Medium-low	16.1% (14.5–17.8%)
		Low	0.8% (0.4–1.2%)
Household income	1902	< €1150/month	7.3% (6.1–8.5%)
		€1151–1800/month	15.1% (13.5–16.7%)
		€1801–2600/month	24.7 (22.7–26.6%)
		> €2600/month	52.9 (50.1–55.2%)
Health condition			
Self-perceived health	1905	Bad	0.6% (0.2–0.9%)
		Moderate	15.7% (14.1–17.4%)
		Medium	53.3% (51.1–55.6%)
		Very good	25.0% (23.1–27.0%)
		Excellent	0.5% (0.4–0.6%)
Visited their family doctor	1905	Yes	25.0% (23.0–26.9%)
Visited a specialized doctor	1905	Yes	14.2% (12.7–15.8%)
Previous surgeries	1905	Yes	75.8% (73.9–77.8%)

95% CI: confidence interval 95%

**Table 2:** Preferences about the provided service and attitudes of the surveyed sample

Variables	N	Measurements	Values (95% CI)
Which professional do you prefer to provide the service?	1905	Anaesthesiologist	65.3% (63.1–67.4%)
		Physician assistant	4.9% (4.0–5.9%)
		Indifferent	29.8% (27.7–31.8%)
Trust in the insurance company 1 = Minimum 10 = Maximum	1783	Mean	4.8 (4.7–4.9)
		Median (IQR)	5 (4–7)
Trust in the Government 1 = Minimum 10 = Maximum	1784	Mean	5.5 (5.3–5.7)
		Median (IQR)	6 (4–7)
Possible strategic bias 1 = Minimum 10 = Maximum	1786	Mean	4.8 (4.7–4.9)
		Median (IQR)	5 (3–6)
Stated propensity to risk (1–10)	1783	Mean	4.1 (4.0–4.2)
		Median (IQR)	4 (3–5)

95% CI: confidence interval 95%

IQR: Interquartile range

The studied sample is representative of a typical population pyramid of a Central European country, with a medium-high socioeconomic level, medium or high educational level, moderate satisfaction about their health condition, and relatively high trust in their government.

Of those who answered the question about WTP (n = 1805), only 36.3% (CI 95%: 34.1–38.6%) provided a positive value to avoid substitution. Of those choosing not to give a WTP value, 68.0% (CI 95%: 65.3–70.7%) stated that this was due to believing that payment for avoiding the substitution should not be questioned, while 22.0% (CI 95%: 19.6–24.4%) answered that they could not afford an extra payment.

Of the subjects answering the question about WTA (n = 1803), only 37.7% (CI 95%: 35.4–39.9%) expressed a positive value to accept compensation for the substitution. Of those not accepting compensation for the substitution, 51.4% (CI 95%: 48.5–54.3%) reported that they could not receive an alternative service and accept compensation for it, whereas 31.7% (CI 95%: 29.0–34.4%) believed they could not accept a payment if the government had made that choice.

Table 3 shows the distribution of WTP, WTA, and intrasubject differences across the whole sample.

**Table 3:** Distribution of WTP, WTA, and disparities in the sample.

	Mean (CI 95%)	Percentile 10	Percentile 25	Percentile 50	Percentile 75	Percentile 90
WTP (€)	268.8 (232.6–305.0)	0	0	0	100	800
WTA (€)	955.2 (792.3–1118.1)	0	0	0	600	3000
Intra-subject difference WTA-WTP (€)	687.2 (521.2–853.2)	− 400	0	0	400	3000

Table 4 presents the results based on our modelling of the distributions of WTP and WTA. It should be noted that the null hypothesis of the correct specification of both models cannot be rejected.

**Table 4:** Inferences from the counterfactual distribution

	Kolmogorov-Smirnov test (p value)	Cramer-von-Misses-Smirnov (p value)
Comparison of WTP model specification (H0 = correct specification)	0.24	0.24
Comparison of WTA model specification (H0 = correct specification)	0.46	0.46
Differences between observed distributions		
H0: No effect found, the difference between quantiles is zero for any chosen quantile	< 0.001	< 0.001
H0: the difference between quantiles is constant and equal to the difference between medians	< 0.001	< 0.001
Stochastic dominance (WTA stochastically dominates WTP)	< 0.001	0.020
Inverse stochastic dominance (WTP stochastically dominates WTA)	0.640	0.640

The remaining test results presented in Table 4 refer to hypotheses on the differences between the distributions of WTP and WTA per Eq. 1 (H0i). The hypothesis of no difference between the quantiles of WTA and WTP was rejected for any chosen quantile, as was the hypothesis that such differences are constant. Furthermore, the null hypothesis of the absence of stochastic dominance was also rejected, which means that, for any monetary value M within the set range, the proportion of subjects reporting values of WTP ≤ M is greater than (or equal to) the proportion of subjects reporting WTA ≤ M.

Table 5 presents the parameter estimates for the conditional quantile functions of the distributions of WTP and WTA for quantiles 70, 80, and 90, and Table 6 shows the contribution of each explanatory variable to the differences between WTP and WTA (absolute values) per equation (8). Subjects older than 65 years were related to smaller gaps between WTA and WTP throughout the whole distribution, with a decrease of 21–27%. So H0_1 was rejected. A lower income level increased the gap between WTA and WTP, explaining 9% of it at the 90th percentile of the distribution, H0_2 was refuted too. Risk averse subjects expressed a higher WTP at the 90th percentile, which was related to a smaller WTA-WTP difference that did not reach significance at a 0.05 p value. In this case, H0_3 could not be rejected.

**Table 5:** Quantile regressions at τ = 0.7 (p70), τ = 0.80 (p80), and τ = 0.90 (p90)

	WTP p70	WTA p70	WTP p80	WTA p80	WTP p90	WTA p90
Woman vs. Man	− 2.66** (1.06)	− 103.13 (60.96)	− 93.75** (28.00)	− 241.67 (169.62)	− 229.49** (67.95)	− 216.44 (232.27)
> 65 years of age	3.00** (1.21)	− 337.5** (68.67)	88.28** (32.00)	− 846.9** (191.48)	328.21** (79.35)	− 1190.4** (265.05)
Married vs. Not married	− 1.25 (1.46)	60.94 (82.16)	− 65.23 (40.05)	88.56 (225.23)	− 196.15** (91.52)	234.25 (302.08)
Medium-high vs. High social class	− 96.67** (1.38)	84.9 (78.95)	− 214.4** (36.80)	10.29 (218.46)	− 126.92 (90.81)	− 71.23 (312.25)
Medium vs. High social class	− 96.67** (1.5)	1.04 (86.02)	− 235.6** (39.32)	− 282.35 (240.25)	− 257.69** (96.27)	− 453.42 (340.57)
Medium-low vs. High social class	− 98.33** (1.72)	145.05 (101.74)	− 266.0** (45.36)	− 141.83 (283.26)	− 401.28** (106.38)	− 572.6 (399.33)
Low vs. High social class	− 101.2** (5.62)	− 126.04 (323.13)	− 280.86 (155.7	− 799.67 (935.82)	− 432.05** (153.47)	− 1897.3* (1139.82)
< €1150 vs > €2600/month	− 2.46 (2.33)	79.43 (129.7)	− 23.05 (64.08)	629.74 (353.04)	− 54.49 (149.13)	798.63* (478.25)
€1150–1800 vs. > €2600 €/month	− 2.84 (1.75)	87.76 (100.32)	− 53.52 (47.47)	357.52 (277.66)	− 41.03 (112.53)	365.75 (393.91)
€1801–2600 vs. > €2600 €/month	− 1.81 (1.34)	78.13 (76.18)	− 60.94* (35.53)	263.56 (211.01)	− 173.08** (86.86)	497.26* (289.95)
Moderate vs. Bad health condition	− 1.9 (5.38)	− 179.43 (346.42)	− 59.77 (159.2)	− 913.4 (954.84)	− 146.15 (389.64)	− 68.49 (1311.36)
Medium vs. Bad health condition	− 1.51 (5.27)	− 201.56 (341.64)	− 48.05 (157.19)	− 1076.8 (942.25)	− 29.49 (385.08)	− 187.67 (1299.95)
Very good vs. Bad health condition	0.65 (5.33)	− 228.13 (344.9)	23.44 (158.68)	− 1176.63 (950.61)	57.69 (388.23)	− 353.42 (1307.84)
Excellent vs. Bad health condition	0.16 (5.71)	− 199.48 (363.08)	− 30.47 (167.33)	− 1165.36 (1003.54)	117.95 (410.74)	− 401.37 (1379.63)
Preference for anaesthesiologist vs. indifferent	101.79** (1.17)	− 101.04 (67.8)	225.39** (30.39)	− 322.88 (189.08)	367.95** (72.35)	− 106.85 (253.18)
Preference for PA vs. indifferent	1.25 (2.49)	− 11.72 (144.48)	13.67 (66.87)	− 188.24 (399.66)	− 34.62 (161.25)	− 195.89 (512.53)
Aversion towards risk score (≤ 4)	0.91 (1.09)	− 42.19 (61.51)	48.44 (28.93)	− 188.40 (171.73)	144.87** (69.74)	− 345.21 (237.42)
Trust in government (1–10)	0.30 (0.27)	− 29.43** (15.48)	3.13 (7.16)	− 166.83** (44.07)	14.10 (17.57)	− 161.64** (58.27)
Possible strategic bias (1–10)	− 1.15** (0.23)	25.52* (13.12)	− 24.22** (6.09)	88.56** (35.84)	− 57.69** (14.77)	123.29** (51.44)
Constant	109.68** (6.17)	876.56** (385.68)	641.80** (178.03)	3745.59** (1068.09)	1303.85** (451.38)	3986.3** (1518.12)
Estimated value	94.85	432.36	324.53	1361.95	782.14	1927.88
Observed value	50	400	200	1000	800	3000

^*^ p < 0.1; **p < 0.05; Standard errors in parentheses

**Table 6:** Percentage contribution of each variable to the predicted changes at τ = 0.7 (p70), τ = 0.80 (p80), and τ = 0.90 (p90)

	Difference WTA-WTP p70 (SE)	Difference WTA-WTP p80 (SE)	Difference WTA-WTP p90 (SE)
Woman vs. Man	− 44.3% (30.3)	− 20.6% (26.7)	1% (19.3)
> 65 years of age	− 27.3%** (9.1)	− 23.7%** (4.7)	− 21.3%** (5.3)
Married vs. Not married	14.2% (15.7)	11.1% (13.8)	17.2% (16.2)
Medium-high vs. High social class	17.8% (14.7)	7.0% (10.7)	1.0% (7.2)
Medium vs. High social class	7.2% (9.3)	− 1.1% (6.6)	− 2.5% (5.8)
Medium-low vs. High social class	11.4% (9.8)	1.8% (5)	− 1.4% (4.4)
Low vs. High social class	− 0.1% (0.6)	− 0.4% (0.3)	− 0.6%** (0.3)
< €1150 vs > €2600/month	1.7% (5.5)	4.4% (3.3)	3.2% (2.2)
€1150–1800 vs. > €2600 €/month	4.1% (6.6)	5.8% (4.6)	3.2% (3.1)
€1801–2600 vs. > €2600 €/month	5.8% (7.0)	7.5% (5.6)	8.6% (3.7)**
Moderate vs. Bad health condition	− 8.3% (69.4)	− 12.7% (27.9)	0.6% (29.4)
Medium vs. Bad health condition	− 31.6% (223.0)	− 51.4% (91.5)	− 4.4% (99.4)
Very good vs. Bad health condition	− 16.9% (103.6)	− 28% (43.1)	− 5.3% (46.6)
Excellent vs. Bad health condition	− 3.1% (22)	− 5.5% (8.6)	− 1.4% (10)
Preference for anaesthesiologist vs. indifferent	− 39.5% (26.3)	− 33.8%** (14.8)	− 16.2% (10.7)
Preference for PA vs. indifferent	− 0.2% (4.1)	− 0.9% (2.3)	− 0.4% (1.4)
Aversion towards risk score (≤ 4)	− 7.7% (13.2)	− 13.4% (12.7)	− 15.3%* (8.4)
Trust in government (1–10)	− 48.7% (31.4)	− 88.0%** (22.4)	− 50.4%** (18)
Possible strategic bias (1–10)	38.3%* (22.6)	51.2%** (18.8)	45.5% ** (17.7)
Constant	227.2% (422.3)	290.9%* (176.5)	139.1% (195.2)
Estimated value	337.5	1037.4	1145.7
Observed value	350	800	2200

^*^p < 0.1; **p < 0.05; Standard Errors (SE) in parentheses

Trust in the government was consistently associated with a reduction in the difference between WTA and WTP of up to 88% at the end of the distribution, H0_4 was refuted. In contrast, a possible strategic bias related to an increase in this difference of up to 51%. Subjects who preferred the MDA expressed a smaller difference between WTA and WTP compared with those who were indifferent (33% lower at the 80th percentile), which was mainly explained by higher WTP values (see Table 5).

## Discussion

This study shows the expressed preferences of a representative sample of the Dutch population about the substitution of the MDA. This sort of disinvestment was not preferred by those in the included sample, as it was noted both through the direct questions and through their expressed WTP and WTA for avoiding or being compensating for the new situation. The proportion of the population that demanded to be compensated for a certain monetary value was greater than the proportion of the population that would pay this monetary value. This result is consistent, regardless of the monetary value considered.

The elements significantly associated with a higher difference between WTA and WTP were related to the possible presence of a strategic bias and lower income levels, whereas being older than 65 years, having trust in the government, and having a preference for an anaesthesiologist decreased this gap.

The effect of these factors on the WTA-WTP gap can be explained within the conceptual frameworks formerly discussed. As described in the literature, the income level partly explains the difference between WTA and WTP. Belonging to the least privileged social classes, a correlate of income, was associated with smaller WTP values but not with changes in WTA.

Previous experience using health services reduced information costs, but no relationship was found between this experience and the gap between WTA and WTP. Recognizing the presence of a possible strategic bias and trust in government, which were associated with higher/lower WTA-WTP differences, could also be related to information costs.

The relationship between the strategic bias and an increase in the difference between WTA and WTP is rather intuitive. If subjects believe they can influence the price or its impact on themselves, they could underestimate the WTP and artificially overestimate the WTA [16, 39, 46]. Strategic bias occurs when the subject premeditatedly offers distorted preferences to influence the decision-making process, as he or she believes that the question will be used to determine a favourable decision over other alternatives or if he or she had expectations about the relative likelihood of those alternatives being selected and delivered [47]. Therefore, in this case, strategic bias could be related to a rejection of the substitution.

On the other hand, citizens’ trust in the government appeared to be related to lower WTA-WTP differences. Trust in the government can manifest as a belief that the government is accomplishing its obligations, which can be a determining factor for supporting their planned policies [34], making it unnecessary to search for further information and hence minimizing information costs. In other research fields, citizens who expressed a higher trust in their government offered higher WTP for government initiatives [35] and needed smaller WTA for complying with government programmes [37]. Therefore, citizens who show high levels of trust in the government would be considering that the agency relationship that occurs between them (principal) and the government (agent) is complete [48]. The Dutch express high trust levels in their government [49], although they show a weaker trust in insurers, which could threaten the acceptability of healthcare policies [50].

The observed decrease in the gap between WTA and WTP associated with high trust in the government could also be explained in reference to prospect theory [30]. This theoretical framework states that the combination of risk aversion and uncertainty results in asymmetric adjustments in WTA and WTP. Some experiments have demonstrated how uncertainty and risk aversion increase WTA-WTP differences. Trust between buyers and sellers could decrease uncertainty and diminish this difference [33]. It has been described how trust in specific institutions reduces perceived risks [38], and the high trust levels in their government expressed by the Dutch population have also been related to a major acceptance of risk [51]. In the presence of risk aversion, in a framework of asymmetric information, trust in institutions would act as a compensating element for both asymmetry and aversion. On the other hand, distrust would amplify the effects that both aversion and asymmetry would have separately. The greater the citizens’ trust in institutions is, the greater they are expected to accept changes in the provision of public services, hence the smaller the gap between WTA and WTP. However, if this trust was low or null, the effect could be the opposite, and the difference between both valuations might increase. As far as we are concerned, this study is the first to introduce a variable representing trust in government in the CV of a healthcare service, which could explain differences between WTA and WTP within the frameworks of both the neoclassical economics theory and the so-called “behavioural economics”. The outcome obtained was highly significant and opens the doors to considering the variable inclusion in further research to confirm or reject its usefulness for valuing the studied subject matter.

We did not find that risk aversion was associated with a higher WTA-WTP difference, as has been postulated [20, 30, 32, 33]. It should be noted that the evolved prospect theory, designated as cumulative prospect theory, proposed that individuals are risk averse over gains and risk seeking over losses and that they tend to overweight low-probability events while underweighting the likelihood of high-probability events. Therefore, Tversky and Kahneman [52] described the fourfold pattern of risk attitudes where individuals could behave as risk-seeking over low-probability gains, risk-averse over high-probability gains, risk-averse over low-probability losses and risk-seeking over high-probability losses. As the presented scenario implied a relatively low surgical risk (mortality of 1/200 within a month), people behaved as risk averse and offered a higher WTP to avoid this risk. However, no correlation was found between risk aversion and WTA, which resulted in a lack of association between self-reported risk aversion and a non-significant decrease in the gap between WTA and WTP. Likewise, preference for the anaesthesiologist in a situation of quantified surgical risk was found to correlate with a significant increase in WTP, which “paradoxically” resulted in a decreased gap between WTA and WTP.

Correlations between age and WTA-WTP disparities can also be interpreted within the framework of risk perception. In this study, subjects older than 65 years expressed a smaller difference between WTA and WTP due to two complementary mechanisms: offering higher WTP and demanding lower WTA. A positive correlation between age and WTP for diverse diagnostic technologies has been observed [53], which could be explained by a greater need for them or a greater perception of risk.

This work has limitations. The large proportion of zero responses hinders the extraction of perceived value from the answers. On the other hand, respondents may have perceived that accepting substitution was socially preferable, so we cannot explore the role of social desirability bias in the WTP-WTA disparity [54]. The data had a high quality level for those referring to the sociodemographic characteristics of the subject, which were collected when constituting the panel. The other information was gathered online by means of a standardized process, but its quality cannot be assured. The "acquiescence or complacency bias" ("yea-saying bias"), which can lead to upwardly biased values of the final assessment [55], could be present, although we tried to minimize it by employing the mechanism of the random starting point.

The results of this study have relevant implications for understanding citizens’ preferences and their potential incorporation in planning healthcare services. Health policy planning should consider these preferences and elicit different ways of expressing them. Understanding and disclosing the population’s preferences to shape health policy is consistent with the principles of good governance and has been shown to improve healthcare efficiency and quality [2]. There is an open debate about including rejection to losses when planning health policies since this affects the allocation of services, by impacting not only cost-efficiency but also society values related to the distribution of health resources [56]. In cost-effectiveness studies, as long the acceptability thresholds are understood as the expression of users’ preferences about the received intervention, the decision-making will be clearly asymmetric if it appears in the northwest quadrant (how much one is willing to pay for a new intervention) or southwest quadrant (what the compensation should amount to for withdrawing it) in the cost-effectiveness plot [24, 39, 57]. The results discussed above have direct implications in this regard. As was shown, the population demanded to be compensated with greater amounts of money than what they would pay to avoid the substitution, so identifying threshold values in the southwest quadrant would have important implications in designing and implementing the disinvestment policies of health services and technologies, especially since the usual arguments have revolved around what the QALY value should be from the perspective of WTP. Additionally, previous studies have shown that both citizens and health policymakers are less likely to lose health-related services than to introduce new ones of similar value. Loss aversion is one of the possible explanations for this result [58]. This not only opens up a new research field but also poses new ethical dilemmas for policymakers in terms of considering symmetric or asymmetric thresholds depending on the cost-effectiveness quadrant where the decision appears.

Another highly important outcome is the identification of a profile of citizens who could reject the substitution by means of the differences of their expressed WTA and WTP [24], whether it is due to their personal characteristics or to beliefs typical of their social background such as a lack of trust in the government. This is of great informational value for designing health services. Additionally, the work carried out by institutions to improve citizens’ confidence must not only be considered an imperative for good governance but also for the consequences it has for the acceptability of already-made decisions. In this sense, another important implication of our result is that the transferability of contingent valuation studies from one country to another is limited not only by differences in per capita income but also by different social and cultural values between citizens of different countries. Thus, the results achieved in this study cannot be extrapolated to those that would be obtained in other countries since trust in their institutions can vary considerably.

## Conclusion

In conclusion, health policymakers must consider that an important group of citizens reports different perceived values of services from the perspectives of gains and losses. This tendency is apparent when assessing a substitution example in the Dutch health system. In the studied population, despite the high proportion of zero responses, two characteristics that accounted for these disparities were pointed out, trust in the government, which can minimize the perception of risk, and acknowledging a possible strategic bias that could express a certain negative attitude towards substitution. Understanding the reasons why citizens refuse to attribute value to health services remains a challenge for researchers and institutions. Nevertheless, identifying a profile of citizens who are averse to losing health services should be considered for both designing and implementing new health services or interventions and in making disinvestment decisions. Trust in public institutions appears to be one characteristic that should be further considered when studying citizens’ preferences in the field of health.

## Supplementary Information

**Additional file 1.** Clinical scenarios and questions to elicit Willingness to pay and Willingness to accept.

## Data Availability

The datasets used and/or analysed during the current study are available from the corresponding author on reasonable request.

## Abbreviations

CI: Confidence interval
CV: Contingent valuation
IQR: Interquartile range
MDA: Medical doctor trained in anaesthesia
PA: Physician assistants
QALY: Quality adjusted life year
WTP: Willingness to pay
WTA: Willingness to accept
