# Supplementary material for: Risk aversion, trust in institutions and contingent valuation of healthcare services: trying to explain the WTA-WTP gap in the Dutch population
Source: Cost Eff Resour Alloc. 2021 May 5;19:27. doi: 10.1186/s12962-021-00281-9 (PMC8097777; doi:10.1186/s12962-021-00281-9)
Supplement: Supplementary file 1 — Additional file 1. Clinical scenarios and questions to elicit Willingness to pay and Willingness to accept. [file 12962_2021_281_MOESM1_ESM.doc]

Appendix 1. Questions to elicit Willingness to pay and Willingness to accept

Introduction

Thank you for participating in this research survey. Through our research, we would like to know the choice and value that citizens assign to a specific service in health care: anesthesia provided by physicians versus anesthesia provided by Physician Assistants.

The survey is preceded by a description of the issue under research. Reading this description will take you about 10 minutes.

Afterwards, you will be presented with two scenarios with questions. Reading the scenarios and answering the questions will take you about 25 minutes.

Description of the service provider: the anesthesiologist

If you need to undergo a painful procedure in a hospital, say a hip replacement surgery or removal of a tumor in your abdomen, you need to be anesthetized. The physician who anesthetizes you is called the anesthesiologist.

The anesthesiologist is part of the surgical team also composed of a surgeon (i.e. the doctor who performs the surgery) and several nurses. The function of the anesthesiologist within the surgical team is to make sure that the patient suffers no pain and that all important functions of the body keep on functioning properly during and after surgery. In a typical surgery, the anesthesiologist will either give a general anesthetic or a so called regional technique to the patient. During a general anesthetic the patient sleeps and is numb to pain during the operation. For a regional technique the anesthesiologist numbs the part of the body to be operated by means of an injection. In practical terms, there is no difference in risk between general anesthetic and a regional anesthetic technique.

Another important function of the anesthesiologist is also to determine potential risks prior to the surgery. In this so-called preoperative screening, the anesthesiologist checks the patient's history and carries out a physical examination to determine how risky a specific surgery can be for the patient. For example, if the patient suffers from diabetes and needs to undergo surgery, the anesthesiologist investigates the condition of important organ such as the heart and kidneys and optimizes them prior to the day of surgery. Thus does The anesthesiologist then prepares an anesthetic plan to counteract the potential effects of surgery for the patient.

After the surgery, you spend time under observation in the so called recovery room. The function of the anesthesiologist in the recovery room is to oversee that your bodily functions function correctly and that your pain level remains acceptably low.

It takes a total of 11 years to become an anesthesiologist: 6 years of university medical studies to become a physician (i.e. Medical Doctor) and 5 years of university specialized education to become an anesthesiologist.

Improved scientific knowledge, education of the practitioners and monitoring of vital functions have contributed to the current very low risks of complications or death due to anesthesia. The risk of someone dying due to anesthesia is estimated to be less than 1 in 250,000 anesthetics. It has been calculated that a per-son is more likely to die struck by a lightning than to die due to an anesthetic. However low this risk seems, it is not always completely absent. This comes from the fact that even a simple surgery is perceived by our body as a form of invasive aggression of the system, many times coupled with tissue damage. Sometimes adverse effects to medication, or an unforeseen situation during surgery such as blood clot, can lead to severe accidents and eventually death.

An alternative for the anesthesiologist: the Physician Assistant

The Dutch government has introduced the physician assistant as an alternative to the anesthesiologist to take part of the workload off anesthesiologists so that the latter can work more efficiently. This can translate into physician assistants increasingly working on their own for example in non-complex surgeries or evaluating patients prior to surgery.

It takes 5,5 years to become a physician assistant. Physician assistants first become anesthesia technicians, a process which takes 3 years of study and practice at the MBO (intermediate level professional education) level, but which in practice is considered a HBO (higher level vocational education or community college level) level study. After anesthesia technicians have completed this first part of their education, they need to practice in a hospital under the supervision of a physician for at least two full years before they undertake a 2.5-year HBO-Master's level training to finally become physician assistant in anesthesiology.

Under normal circumstances, the introduction of a new drug, intervention or service for the treatment of patients is preceded by rigorous research (for example clinical trials). This is a long process which normally takes around 20 years. In general terms, a drug, intervention or service needs to be at least as safe as the already existing alternatives. It also needs to be specific. For example, e.g. a new drug is tested and approved safe only for a specific disease, despite the similarities with other existing drugs that might be approved for the same disease. In the case of physician assistants, such studies and safety checks have not been carried out prior to their introduction as health care professionals substituting the anesthesiologists in some of his or her tasks, including the operation room work.

In this scenario you are a healthy 55-year old person. Since a few weeks you have obstipation problems that cause you bouts of belly ache. The pain is at times severe (7 or higher on a scale from 0= no pain, to 10= the worst imaginable pain).

You visit your family physician and he refers you to the hospital for further tests, among others a scan of your abdomen and a endoscopy of your intestines. When the results are back, your family physician explains that you have a tumor in your large intestine, and that the pain was the result of partial obstruction of stool passage. The tumor has apparently not been disseminated, however you need to have the tumor removed by a surgeon.

The surgeon tells you that the tumor should be removed. In order to avoid the dissemination of the tumor to other parts of the body, the surgeon advices to operate within 6 weeks. The risk associated with dissemination of the tumor to other parts of the body is that your chances of being alive after five years drop substantially (from a 73% survival rate after 5 years for a cancer that remains local, to 6% if the cancer has spread to other organs). The surgeon explains that the operation will consist of removing the left side of your large intestine and it is called a Hartmann procedure.

For a Hartmann procedure, the Dalton predicted risk of for a healthy 55-year old within 30 days after surgery is 0,5%. This is the same as saying that 1 out of 200 patients will probably die within 30 days after a Hartman procedure.

Due to shortage of anesthesiologists, you are given two options: either be operated tomorrow with the anesthetic delivered by a physician assistant, or wait for an anesthesiologist to be available, or pay an extra fee to have an anesthesiologist take care of you during the Hartmann procedure.

Would you pay for an extra amount of money to assure the presence of an anesthesiologist during your Hartmann procedure (Dalton 30-day probability of death of 0,5% and increasing risk of extension of the tumor to other organs). (Remember there is no right or wrong answer. The value you indicate is what is important to us).

No, because:

- - - I cannot afford to dedicate more financial resources towards my health care insurance policy;
    - I do not agree with the fact of having to pay more for something which was part of the standard package until now;
    - Another reason: ...

Yes: How much (expressed in euro) would you pay to assure you the availability of an anesthesiologist during the operation?

Would you pay an extra charge of 25 €?                  □Yes □ No

If yes, would you pay an extra charge of 50€?                    □Yes □ No

If yes, would you pay an extra charge of 100€?                  □Yes □ No

If yes, would you pay an extra charge of 200€?                 □Yes □ No

If yes, would you pay an extra charge of 400€?                  □Yes □ No

If yes, would you pay an extra charge of 600€?                  □Yes □ No

If yes, would you pay an extra charge of 800€?                  □Yes □ No

If yes, would you pay an extra charge of 1000€?                □Yes □ No

If yes, would you pay an extra charge of 1500€?                □Yes □ No

If yes, would you pay an extra charge of 2000€?                □Yes □ No

If yes, would you pay an extra charge of 3000€?                □Yes □ No

If yes , how much maximum extra charge would you pay? (THIS ONE LEAVE OPEN ENDED)

Are you completely sure that if you were in reality faced with this condition, you would pay the amount of money you have mentioned?

- Yes
- No : I would pay … euro

Now imagine that due to a shortage of anesthesiologists the insurance company offers you the following deal: since you will not be anesthetized by an anesthesiologist (as is agreed in your insurance package) but by a physician assistant, the insurance company offers to compensate you with a sum of money. Would you accept money and forego the service of an anesthesiologist, and in that case which minimum amount of money would you accept as compensation? (Remember there is no right or wrong answer. The value you indicate is what is important to us).

Yes. What is the minimum amount of money that you would request as compensation?

Would you feel compensated if you received 25 euros? □Yes □ No

If not, would you feel compensated if you received 50€? □Yes □ No

If not, would you feel compensated if you received 100€? □Yes □ No

If not, would you feel compensated if you received 200€? □Yes □ No

If not, would you feel compensated if you received 400€? □Yes □ No

If not, would you feel compensated if you received 600€? □Yes □ No

If not, would you feel compensated if you received 800€? □Yes □ No

If not, would you feel compensated if you received 1000€? □Yes □ No

If not, would you feel compensated if you received 1500€? □Yes □ No

If not, would you feel compensated if you received 2000€? □Yes □ No

If not, would you feel compensated if you received 3000€? □Yes □ No

If not, how much minimum amount of money should you receive to feel compensated? ...€

No, I would not accept money because :

- - - I do not perceive any differences between anesthesiologists and physician assistants and thus do not need to be compensated for anything.
    - Since the government has decided physician assistants are good enough to do the job, I trust that decision.
    - I would not accept not having an anesthesiologist, and thus not undergo an anesthetic in the Netherlands.

Imagine, however, that there was no option and that you would get the *physician assistant* anyway and that you were told that you would get the money from the insurance company anyway in the amount of 3000 Euro. What would you do with the money?

- Keep it and
  - Use it for my own expenses
  - Use it towards lowering my insurance premium
- Donate it
- So that it could be put to better use in the health care system
  - To a good purpose like orphanages, cancer research, etc.

(IF YES): Are you completely sure that if you were in reality faced with this condition, you would request the amount of money you have mentioned?

- Yes
- No : I would request … euro
